# Sensitivity of a Label-Free Guided-Mode Resonant Optical Biosensor with Different Modes

**DOI:** 10.3390/s120709791

**Published:** 2012-07-18

**Authors:** Qi Wang, Dawei Zhang, Huiyin Yang, Chunxian Tao, Yuanshen Huang, Songlin Zhuang, Ting Mei

**Affiliations:** 1 Engineering Research Center of Optical Instruments and Systems, Ministry of Education, Shanghai Key Lab of Modern Optical Systems, University of Shanghai for Science and Technology, No. 516 Jungong Road, Shanghai 200093, China; E-Mails: shelly3030@163.com (Q.W.); yanghuiying2352@sina.com (H.Y.); chxtao@live.com (C.T.); hyshyq@sina.com (Y.H.); slzhuangx@yahoo.com.cn (S.Z.); 2 Laboratory of Nanophotonic Functional Materials and Devices, Institute of Optoelectronic Materials and Technology, South China Normal University, Guangzhou 510631, China; E-Mail: ting.mei@ieee.org

**Keywords:** sensitivity, biosensor, TE mode, TM mode, guided mode resonance (GMRF)

## Abstract

Sensitivity is a key factor in the performance of a sensor. To achieve maximum guided-mode resonant optical biosensor sensitivity, a comparison of biosensor sensitivity for Transverse Electric (TE) and Transverse Magnetic (TM) modes based on the distribution of electric fields is presented in this article. A label-free guided-mode resonant optical biosensor is designed using the quarter-wave anti-reflection method to reflect only a narrow band of wavelengths modulated by the adsorption of a biochemical material on the sensor surface at the reflected frequency. With the distribution of electric fields simulated according to the Rigorous Coupled Wave Analysis (RCWA) theory, it is found that the full width at half maximum of the TM mode is (∼4 nm) narrower than that of the TE mode (∼20 nm), and the surface sensitivity of the TE mode incident light is three times that of the TM mode. It is proposed in this article that the light mode plays an important role in the sensitivity of guided-mode resonant biosensors.

## 1. Introduction

Research on label-free guided-mode resonant (GMR) optical biosensors has recently attracted great interest owing to applications for detecting a variety of bio-molecular complexes such as oligonucleotides, antibody-antigens, and enzyme-substrate interactions. A GMR optical biosensor comprised of a surface relief sub-wavelength grating is designed to reflect only a single wavelength while illuminated with white light [1]. Utilizing the GMR effect for sensor applications was first suggested by Magnusson and Wang in 1992 [2,3]. In comparison to classical input or output grating couplers, GMR optical biosensors differ distinctly from traditional sensors in their operational principle and functionality [4,5]. Wawro *et al.* presented a method using fiber optic sensor integrating dielectric diffraction gratings and thin films on optical fiber endfaces for biomedical sensing applications [6]. In2002, Cunningham *et al.* discussed the use of these resonant elements as biosensors, capable of resolving changes of 0.1 nm in a sample. They also make it possible to quickly measure a large number of molecular interactions taking place simultaneously upon a grating surface. Moreover, they can monitor reactions in real time [7–10]. Furthermore, Cunningham *et al.* improved the sensitivity by changing the GMR structure [8]. The polarization of the incident light is a key factor in the sensitivity of this kind of optical biosensor. Magnusson *et al.* emphasized polarization-based parametric discrimination and presented that resonant sensors can be designed to support two or more leaky modes in the spectral band of interest [11]. Electric field distribution analysis (EFDA) can reveal the difference the Transverse Electric (TE) and Transverse Magnetic (TM) modes have on sensitivity. This article presents a comparison of sensitivity in the TE and TM modes using EFDA. It is found that sensitivity in the TM mode is three times that achieved in the TE mode.

Magnusson and Lee introduced a phase modulation method [12,13], which is more complex than the amplitude modulation method, so the amplitude method is used in this article. When molecules are attached to the surface, the reflected wavelength (color) is shifted due to a change in the optical path of light that is coupled into the grating. In this article, the appropriate parameters of a GMR biosensor structure are determined and the sensitivity of the different modes of incident light is shown. Then the sensitivity of the TE and TM modes of incident light is compared by the EFDA.

## 2. Model

**Figure 1.**
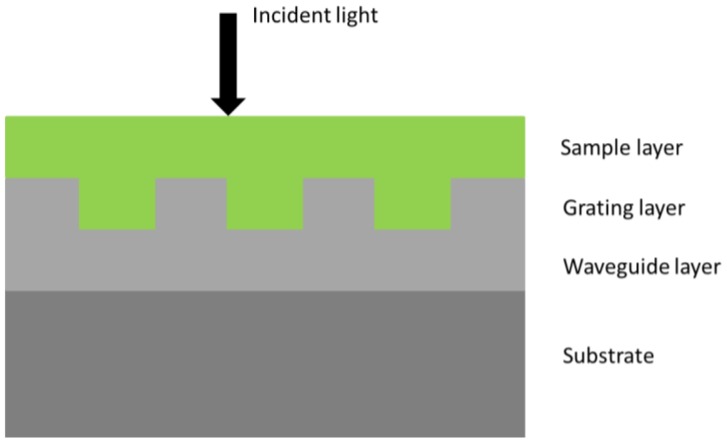
Structure of the GMRF The parameters are n_h_ = n_w_ = 1.98 (HfO_2_), n_l_ = n_c_ = 1.0 (air), n_s_ = 1.52 (quartz glass), Λ = 500 nm, d_w_ = 101 nm, d_g_ = 120 nm.

The structure of the GMR optical biosensor is shown in Figure 1. From top to bottom, the GMR optical biosensor includes a cover layer (air), a sample layer (biological samples, such as protein molecules), a grating layer, a waveguide layer, and a substrate layer (quartz glass). Based on the theory of the optical waveguide, the effective index of the i-th order diffracted wave should be in the range followed with Equation (1) in [14]:

(1) $$\text{max}\{n_{c},n_{s}\}\leq \left|\beta_{i}/k_{0}\right|=\left|n_{c}\text{sin}\theta -i\lambda /\Lambda \right|<\text{max}\{n_{m}|m=1,2,3\ldots \}$$

Here, n_c_ and n_s_ are the refractive index of the sample layer and the substrate, β is the propagation constant, k_0_ is the wave number, θ is the incident angle, λ is the resonant wavelength, and Λ is the period of the grating layer. Only to abide by Equation (1), the phenomenon of guided mode resonance effect could be observed. Meanwhile, the position of the resonant wavelength can be obtained.

In our experiment, the structure parameters are as follows: the refraction index of the grating layer is n_h_ = 1.98 (HfO_2_); n_l_ = n_c_ = 1.0 (air); the grating period Λ is 500 nm; the substrate refraction index n_s_ is 1.52 (quartz glass); the refraction index of the waveguide layer is n_w_ = 1.98 (HfO_2_). According to these parameters, the range of the resonant wavelength we got to be 760 < λ < 990. Here, we choose λ = 800 nm. The anti-reflective conditions require the thicknesses of the waveguide layer and the grating layer to be one quarter-wavelength to optimize the structure. The thickness of the waveguide layer d_w_ is 101 nm and the grating layer d_g_ is 120 nm. At this point, all the structure parameters of the GMRF biosensor have been determined and are summarized in Table 1.

## 3. Results and Discussion

**Figure 2.**
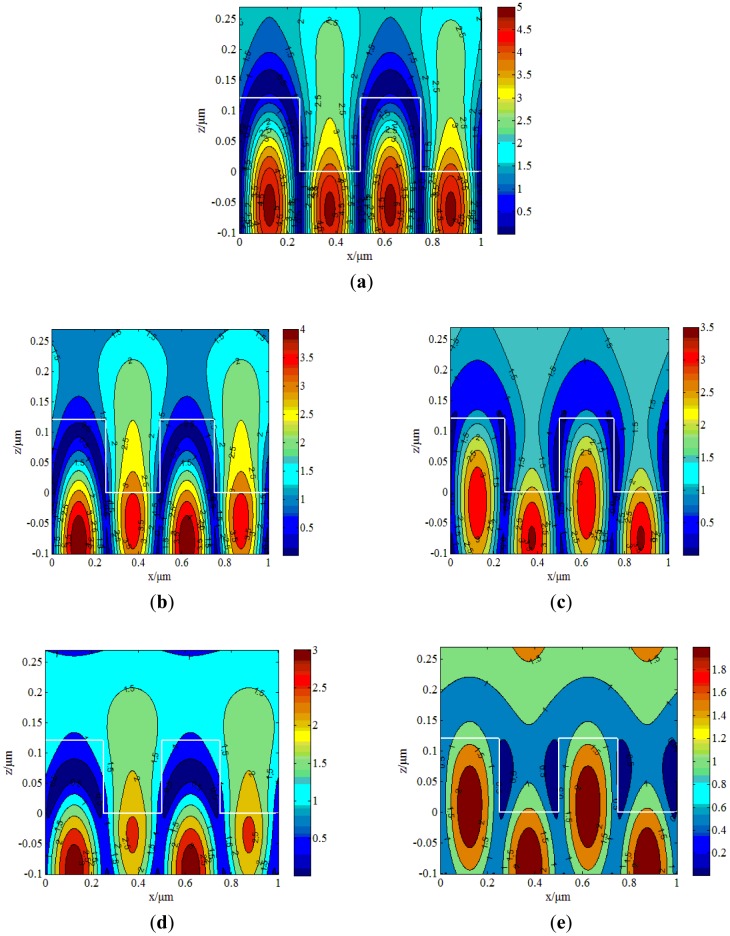
Electric field distribution of TE mode at different wavelengths. (**a**) Electric field distribution at the wavelength of 798 nm with TE mode incident into the GMRF. (**b**) Wavelength at 788 nm. (**c**) Wavelength at 808 nm. (**d**) Wavelength at 778 nm. (**e**) Wavelength at 818 nm.

Based on the GMR structure we designed in the previous section, the electric field distribution can be obtained using the RCWA method. Here, a comparison of biosensor sensitivity for the TE and TM modes based on the distribution of electric fields is presented. According to the RCWA method, the resonant wavelength of incident light in the TE mode is 798 nm without a biological sample attached on the surface, while the resonant wavelength of the TM mode is 766 nm. Because the sensitivity of this biosensor is related to the full width at half maximum (FWHM), FWHM should be discussed. The FWHM can be observed via the electric field distribution. Figure 2(a) presents the electric field distribution of the TE mode for the resonant wavelength of 798 nm. We can see that the strongest electric field excited in the waveguide layer is 5times greater than that in the weakest part of the field. When the resonant wavelength shifts 10 nm, which is from 798 nm to 788 nm or 808 nm, the electric field distributions are shown in Figure 2(b,c), respectively, which still show a strong guided-mode resonance effect where the strongest part is 4 to 3.5 times greater than the weakest part of the field. With the wavelength changed by 20 nm to 778 nm and 818 nm, the electric field distributions are shown in Figure 2(d,e), respectively. However, the GMR effect is very weak. In particular, at the wavelength of 818 nm in Figure 2(e), it is difficult to discern any guided-mode resonance effect.

**Figure 3.**
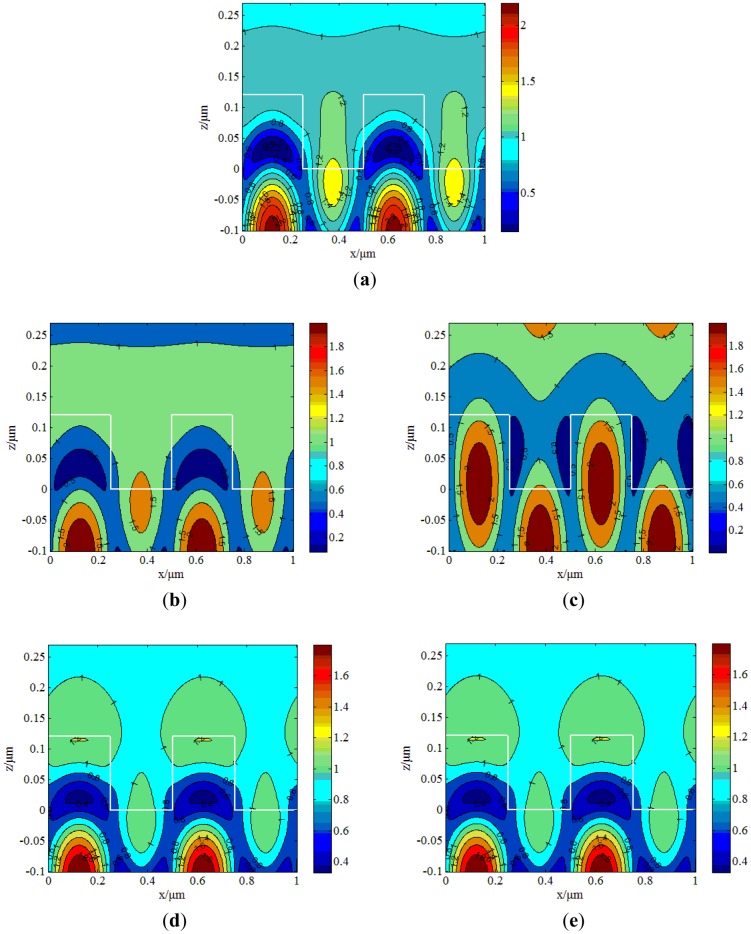
Electric field distribution of TM mode at different wavelengths. (**a**) Electric field distribution at the wavelength of 766 nm with TM mode incident into the GMRF. (**b**) Wavelength at 763 nm. (**c**) Wavelength at 769 nm. (**d**) Wavelength at 760 nm. (**e**) Wavelength at 772 nm.

In contrast, when illuminated with incident light in the TM mode, the electric field distribution calculated by the RCWA method is shown in Figure 3.

The resonant wavelength for the TM mode is 766 nm. Similarly, incident light is only changed 3 nm, to 763 nm and 769 nm, and the corresponding electric field distributions, shown in Figure 3(b,c), respectively, change slowly. Figure 3(d,e) shows the electric field distribution at 760 nm and 772 nm, respectively, which have sharp electric field distribution changes, but exhibit lower intensity. Using the RCWA method, the FWHM for the TE mode is ∼20 nm while the TM mode's FWHM is ∼4 nm. Due to this contrast, the conclusion is that the energy distribution of the TM mode is more concentrated than the TE mode, so the TM mode is more sensitive than the TE mode.

The different electric field distribution for TE and TM mode is due to the different waveguide eigenvalue equations for different polarization cases. If the effective mode propagation constant of the i-th order evanescent diffracted wave in the waveguide grating is given by:

(2) $$\beta_{i,v}=k_{0}(n_{c}\sin\theta -i\lambda /\Lambda )$$

The mode propagation constant can be obtained by solving the classical eigenvalue equation for the homogeneous slab waveguide given by [15]:

(3) $$\tan(k_{i,v}d)=\frac{k_{i,v}(\gamma_{i,v}+\delta_{i,v})}{k_{i,v}^{2}-\gamma_{i,v}\delta_{i,v}}$$

where the Z-components of the wave number in the cover, grating, and substrate regions are described by:

(4) $$\gamma_{i,v}={\left(\beta_{i,v}^{2}-n_{c}^{2}k_{0}^{2}\right)}^{1/2},\kappa_{i,v}={\left(n_{av}^{2}k_{0}^{2}-\beta_{i,v}^{2}\right)}^{1/2},\delta_{i,v}={\left(\beta_{i,v}^{2}-n_{s}^{2}k_{0}^{2}\right)}^{1/2}$$

respectively. A similar argument applies for the TM polarization case, and thus the waveguide eigenvalue equation for the TM polarization case is given by:

(5) $$\tan(k_{i,v}d)=\frac{n_{av}^{2}k_{i,v}(n_{s}^{2}\gamma_{i,v}+n_{c}^{2}\delta_{i,v})}{n_{c}^{2}n_{s}^{2}k_{i,v}^{2}-n_{av}^{4}\gamma_{i,v}\delta_{i,v}}$$

where the according refractive index of cover layer, substrate layer and grating layer are denoted as *n_c_*, *n_s_*, $n_{av}=\sqrt{fn_{H}^{2}+(1-f)n_{L}^{2}}$ for TE polarization, while $n_{av}=n_{H}n_{L}/\sqrt{n_{L}^{2}f+n_{H}^{2}(1-f)}$ for TM polarization.

Using water (n = 1.333), isopropanol (n = 1.377), and methylsulfoxide (n = 1.479) as the biological samples, which are coated on the surface of the GMR biosensor, the resonant wavelengths of the TE mode obtained by the RCWA method are 807 nm, 811 nm, and 818 nm, respectively, and the TM mode resonant wavelengths are 796 nm, 813 nm, and 826 nm, respectively. Tables 2 and 3 show the resonant wavelength and the resonant wavelength shifts with different samples.

Sensitivity of the biosensor can be obtained from Equation (6) in [16]:

(6) $$S_{n}=\partial \lambda /\partial \text{n}$$

**Figure 4.**
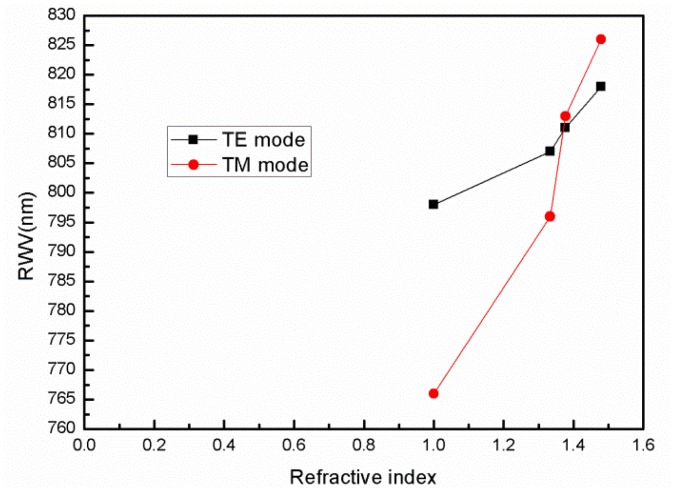
Sensitivity of the TE mode and TM mode.

According to Equation (6), the sensitivity of the TM mode is 113 nm/RIU, which is more sensitive than the TE mode (34 nm/RIU). Figure 4 shows that the sensitivity of the TM mode changes more quickly with refractive index than the TE mode, which indicates that the TM mode is more sensitive than the TE mode. Comparing Figure 1(a) and Figure 2(a), we find that with the TM mode electric field excited in the grating structure has less intensity than that of the TE mode, but has more sensitivity. When the resonant wavelength is incident into the grating, almost all of the energy is coupled into the waveguide layer [17,18]. The strongest part is 5 times stronger than the weakest part for the TE mode as shown in Figure 2(a), while for TM case is only 2.2. The structure designed in this article is to match the resonant wavelength. When the matching wavelength is incident into the gating, the energy will redistribute such that almost all the energy is coupled into the waveguide layer.

## 4. Conclusions

The sensitivity of a GMR biosensor was theoretically analyzed by the electric field distribution. Using the method of quarter wave-length anti-reflection, a GMR biosensor structure was designed in this article. Then the incident light in the TE and TM modes was analyzed by the RCWA method. Through the electric field distribution, it is found that FWHM of the TM mode is narrower than that of the TE mode. In the waveguide layer, the electric field amplitude for TM mode at the resonant wavelength is less intense than that of TE mode. The energy distribution of TM mode is much more concentrated than the TE mode, which means that the TM mode is more sensitive than the TE mode. It can be concluded that the light modes play the important role on the sensitivity of guided-mode resonant biosensors. For the same structure GMR biosensor, using the TM mode will enhance the sensitivity of this optical biosensor. To improve the accuracy of the GMR biosensor, using the TM mode is the best choice.

## Figures and Tables

**Table 1.** Parameters of the GMR biosensor.

**n_c_**	**n_s_**	**n_H_**	**n_L_**	**n_w_**	**d_g_**	**d_w_**	**f**	**λ**	**θ**	**_Λ_**
1.0	1.52	1.98	1.0	1.98	120 nm	101 nm	0.5	800 nm	0°	500 nm

**Table 2.** Resonant wavelength of different samples with TE mode.

**Refractive index**	**1.0 (air)**	**1.333 (water)**	**1.377 (isopropanol)**	**1.479 (methylsulfoxide)**
Resonant wavelength (nm)	798	807	811	818
Resonant wavelength shift (nm)		9	13	20

**Table 3.** Resonant wavelength of different samples with TM mode.

**Refractive index**	**1.0 (air)**	**1.333 (water)**	**1.377 (isopropanol)**	**1.479 (methylsulfoxide)**
Resonant wavelength (nm)	766	796	813	826
Resonant wavelength shift (nm)		30	47	60
